# Functional and Structural Correlates of Motor Speed in the Cerebellar Anterior Lobe

**DOI:** 10.1371/journal.pone.0096871

**Published:** 2014-05-06

**Authors:** Uwe Wenzel, Marco Taubert, Patrick Ragert, Jürgen Krug, Arno Villringer

**Affiliations:** 1 Institute of Training Science and General Kinesiology, University of Leipzig, Leipzig, Germany; 2 Max Planck Institute for Human Cognitive and Brain Sciences, Leipzig, Germany; University of Electronic Science and Technology of China, China

## Abstract

In athletics, motor performance is determined by different abilities such as technique, endurance, strength and speed. Based on animal studies, motor speed is thought to be encoded in the basal ganglia, sensorimotor cortex and the cerebellum. The question arises whether there is a unique structural feature in the human brain, which allows “power athletes” to perform a simple foot movement significantly faster than “endurance athletes”. We acquired structural and functional brain imaging data from 32 track-and-field athletes. The study comprised of 16 “power athletes” requiring high speed foot movements (sprinters, jumpers, throwers) and 16 endurance athletes (distance runners) which in contrast do not require as high speed foot movements. Functional magnetic resonance imaging (fMRI) was used to identify speed specific regions of interest in the brain during fast and slow foot movements. Anatomical MRI scans were performed to assess structural grey matter volume differences between athletes groups (voxel based morphometry). We tested maximum movement velocity of plantarflexion (PF-V_max_) and acquired electromyographical activity of the lateral and medial gastrocnemius muscle. Behaviourally, a significant difference between the two groups of athletes was noted in PF-V_max_ and fMRI indicates that fast plantarflexions are accompanied by increased activity in the cerebellar anterior lobe. The same region indicates increased grey matter volume for the power athletes compared to the endurance counterparts. Our results suggest that speed-specific neuro-functional and -structural differences exist between power and endurance athletes in the peripheral and central nervous system.

## Introduction

Up to date Magnetic resonance imaging (MRI) has successfully been used to identify the function and structure of the human brain in dependence of motor learning and level of skills [1]. In doing so, tasks with high demands of coordinative abilities (e.g. playing piano, juggling, dynamic balancing task, gymnastics) were used to study training-related brain plasticity. Surprisingly little is known about the influence of physical abilities like endurance or speed on functional and structural brain alterations in the field of sport. It is known that for achieving extraordinary speed and power a relatively high discharge rate of motoneurons is necessary to activate as many fast-twitch-fibres (FT-fibres) as possible [2], [3], which in turn are beneficial in producing rapid movements [4], [5]. The question arises in which brain regions these discharge patterns are generated and whether there is a unique structural feature that enables the corresponding firing frequencies, and in return provides the ability for power athletes to excel and execute on a very high level.

There is evidence that the discharge rate of pyramidal tract neurons correlates with the movement velocity of the monkey's forelimb [6]. Furthermore the precentral gyrus is discussed to encode speed information [7]. Turner and colleagues (2003) report that the activity in the basal ganglia, sensorimotor cortex and cerebellum is modulated as a function of speed, where in particular the cerebellum seems to play a major role [8], [9]. Indeed, the cerebellum is characterised as the neural site which encodes speed information and provides a forward internal model to plan or control movements in a kinematic framework [10]. According to this, several animal studies observed correlations between discharge rate of Purkinje cells and movement velocity [11], [12]. In humans, it could be shown that patients with cerebellar lesions are not able to generate fast arm speeds compared to healthy controls [13].

Endurance and power training lead to versatile adaptations of the neuromuscular system [14], [15]. In particular, power training increases the discharge rate of motor units [16], which in turn influences the portion of fast- and slow-twitch-fibres [17], [18]. However, to the best of our knowledge, structural brain differences between power and endurance athletes remain complete speculation. Nevertheless, a number of studies identified structural differences in skilled performers as compared to non-skilled subjects [19]–[22]. In addition, even the adult brain indicates a remarkable capacity for morphological and functional adaptations following different kinds of motor training [23], [24].

It is thought that the neuronal discharge rate is a result of temporal and spatial summation of action potentials at the dendritic tree [25]. Since the latter is bound to the grey matter volume, we hypothesized that a group of power athletes will present (1) superior movement velocity, (2) higher discharge rate of motoneurons and (3) higher grey matter volume of speed specific brain regions such as (sensori-) motor cortex, basal ganglia and Cerebellum.

## Materials and Methods

### Subjects

Due to the continuous demand of producing quick muscle contractions it is no surprise that power athletes show superior performance in speed and power tests (e.g. drop & squat jump) compared to their endurance counterparts [26], [27]. The current study investigated thirty-two healthy track-and-field athletes from regional to international level after obtaining written informed consent. The study was performed in accordance with the declaration of Helsinki as well as approved by the local ethics committee of the University of Leipzig. According to the expected motor speed, all middle- and long-distance runners were allocated to the endurance group (n = 16, age  = 26.5±3.62 years, body height  = 173.7±9.16 cm, body weight  = 68.9±10.75 kg, years of training  = 9.4±6.05, 5 females) and all sprinters, jumpers and throwers were allocated to the power group (n = 16, age  = 23.6±3.91 years, body height  = 182.6±6.72 cm, body weight  = 77.2±9.71 kg, years of training  = 12.5±4.13, 3 females). Since there are significant differences in age (t = 2.21, df = 30, p = 0.04), body height (t = −3.15, df = 30, p = 0.00) and body weight (t = −2.29, df = 30, p = 0.03), we integrated these variables as nuisance covariates in the statistical model described below.

### Experimental overview

The subjects were asked to perform an elementary speed test to obtain maximum movement velocity of the left foot during voluntarily initiated plantarflexion (PF-Test). During task performance electromyographical (EMG) activity of the lateral and medial gastrocnemius muscle was continuously acquired to assess firing frequency of motoneurons, recruitment and selection of fibre types via frequency analysis [28]. All athletes completed a magnetic resonance imaging (MRI) scan of the whole brain on a separate day. The scan involved functional MRI (fMRI) to identify speed specific regions of interest (functional localizer; ROI) and T1-weighted scans to assess structural grey matter volume.

### PF-Test

After a standardized warm-up the subjects were asked to lay prone on an examination table with the ankles hanging over the edge. A light wooden plate with reflecting markers and a 3D-acceleration sensor was attached to the left sole of foot. The foot was still able to move freely and no additional load was applied. The task was to perform the quickest possible plantarflexion from a dorsiflexed position. Task instructions were repeated two to three times before three attempts with full measurements were performed. Two Highspeed-cameras (MIKROTRON, 300 Hz) were adjusted to the left shank to record the movement from a lateral and caudal point of view. The reflecting marker which was used for obtaining maximum velocity (PF-v_max_) was individually set 15 cm from the ankle's centre of rotation in projection of the Chopart-Lisfranc joint. In doing so, we minimized the impact of biomechanical differences (length of calcaneus) which otherwise might have influenced measurable motor speed. The 3D movement analysis was performed using SimiMotion (Simi Reality Motion Systems GmbH, Unterschleiβheim, Germany; http://www.simi.com).

### Surface EMG

EMG activity of the lateral and medial gastrocnemius was measured during task performance using Ag-AgCl surface electrodes (AMBU Blue Sensor M). First, the skin was dry shaved and then cleaned with an alcohol solution to remove hair, grease and loose epithelia. Electrodes were then set in a bipolar configuration parallel to the muscle fibres on the longitudinal midline of the muscles according to the recommendations of SENIAM (http://www.seniam.org). A reference electrode was attached to the tibia. The raw EMG activity was acquired with the Telemyo DTS-system (NORAXON U.S.A. Inc., Scottsdale, Arizona, USA; http://www.noraxon.com) at a sampling frequency of 1500 Hz. Maximum voluntary contractions were used to review signal-transmission and amplifier-calibration. Signals from the EMG amplifier and the acceleration-sensor were transmitted on a continuous basis to a personal computer equipped with the software package MyoResearch 2000. Data analysis was performed off-line using MyoResearch-XP 1.07 Master Edition. The data set was carefully screened for artefacts and crosstalk and the acceleration-time-course was used to identify onset of plantarflexion. Time frames of the first 150 ms were cut, which covered approximately 2/3 of plantarflexion. In doing so, we place the relevant time frame in the phase of positive acceleration and eliminate the slowdown phase. Over each time frame, the median frequency was calculated using the Fast-Fourier transformation integrated in the analysing software.

### Statistical analysis of behavioural and EMG data

Population normality and equality of variances were assessed using Shapiro-Wilk test and Levene's test. Since the t-test maintains a small power advantage when population normality is nearly met [29], the between group differences in mean values were evaluated by using Student's t-test for unpaired data, though the standard normal distribution of PF-v_max_ was not given. A level of p≤0.05 was considered significant. Relative effect sizes (Cohen's *r*) were calculated to judge the practical significance. A level of *r*≥0.3 and *r*≥0.5 was considered to be a moderate and high effect size respectively. Since standard normal distribution was not given, coefficients of correlation were calculated according to Spearman (p≤0.05).

### MRI of the brain

MR imaging data was acquired on a 3 Tesla Magnetom Tim Trio scanner (Siemens) using a 32 channel head coil. The head was fixed with a Tempur-cushion and the left foot was raised by a soft heightening beneath the calf. To analyse speed-specific differences in grey matter volume between power and endurance athletes, T1-weighted images were acquired using a MPRAGE (magnetization-prepared rapid acquisition gradient echo) sequence (repetition time (TR) = 1.3 s, echo time (TE) = 3.46 ms, flip angle  = 10°, field of view (FOV) = 256×240 mm, 176 sagittal slices, voxel size  = 1×1×1.5 mm). Speed-specific brain regions were identified with a functional localizer using fMRI. After the structural scans, subjects were asked to perform visually-paced slow and fast foot movements during fMRI acquisition. Therefore, a slow and a fast movement condition of repeatedly dorsi- and plantarflexions with the left foot were performed. Each condition consisted of 5 time sequences, lasting 30 s each with 30 s rest in between. In this block design the slow condition was followed by the fast. Plantarflexions were externally paced (visual metronome on the screen) with a frequency of 0.5 Hz. The slow and fast conditions were colour-coded with a cross-hair on the screen (white cross-hair for slow and red cross-hair for fast condition). The only difference between conditions was the movement speed of plantarflexions whereas the velocity of dorsiflexions remained comparably slow. Functional images were obtained using echo planar imaging (repetitions  = 302 [151 repetitions/condition], voxel dimension  = 3×3×4 mm, 34 slices, TR = 2000 ms, TE = 30 ms, flip angle 90°, bandwidth  = 1825 Hz). The overall acquisition time was approximately 25 minutes.

### MRI data processing and analysis

Pre-processing of functional images was performed using SPM 5 (Wellcome Trust Centre for Neuroimaging, University College London, London, UK; http://www.fil.ion.ucl.ac.uk/spm) operating under a Matlab environment. Standardroutines (realignment, registration, normalization, smoothing) and default parameters were applied for data preprocessing. Functional scans of each individual were realigned to correct for movement artefacts. Framewise displacement (FD) was calculated according to Power and colleagues (2012) and checked for differences between power and endurance athletes using Student's t-test for unpaired data [30]. No group differences could be found (FD_power_ = 77.62±21.29, FD_endurance_ = 74.34±20.56, p = 0.66). Individual activity maps for slow and fast condition were created using first-level analysis in SPM. Individual maps were normalized to standard Montreal Neurological Institute (MNI) space. Functional images were subsequently smoothed using a Full-width-at-half-maximum (FWHM) Gaussian kernel 6×6×6 mm. In the second-level analysis, one sample t-tests were used to identify significant brain activity for the slow as well as for the fast condition compared to the resting state. To localize speed-specific brain regions, significantly increased activity in the fast condition as compared to the slow condition was identified in a paired t-test. In addition, a 2×2 ANOVA was conducted using SPM 8 with a full factorial design to investigate effects between factors condition (fast vs. slow) and group (power vs. endurance). Speed-specific regions were expected across the whole brain. Therefore, whole-brain family-wise error (FWE) correction at the voxel-level was applied to correct for type 1 error. A voxel was considered significant when p≤0.05.

Pre-processing of T1-weighted images was performed using SPM 8 and VBM 8 Toolbox (http://dbm.neuro.uni-jena.de/vbm.html). Default parameters and standard routines were applied. Images were segmented into grey and white matter and cerebrospinal fluid as well as registered, normalized and modulated according to the DARTEL procedure [31]. During normalization the correction of individual brain-volumes was performed using a non-linear modulation. According to our hypothesis, statistical analysis was restricted to grey matter volume. Thereby, grey matter segments were smoothed with a Gaussian kernel of 8 mm full width at half maximum. Using a two-sample t-test, we compared structural differences in grey matter volume between power and endurance athletes. To correct for differences in age, body height, body weight, sex and intracranial volume, these variables were integrated as nuisance covariates in the statistical model. To look for speed-specific structural differences between groups, the search volume was restricted to the cluster identified in the fMRI-localizer test, contrasting fast minus slow movements. The identified region in the anterior cerebellum was defined as the region of interest (ROI_Cereb_) with a 10 mm radius sphere around the peak voxel. Using fMRI during fast foot movements only, two other ROI's were specified by the local maxima in the motor cortex (ROI_M1_ & ROI_SMA_). Within the ROI's, significant differences were identified using a voxel-level FWE correction. A voxel was considered significant when p≤.05.

Furthermore, we analysed the ROI's using the MarsBar toolbox to estimate the individual mean volume of grey matter. These data were exported to “IBM SPSS Statistics 19” and the relationship to the maximum velocity of plantarflexion was calculated (Spearmans coefficient of correlation; p≤0.05).

## Results

### Behavioural and electromyographical (EMG) results

**Figure 1:**
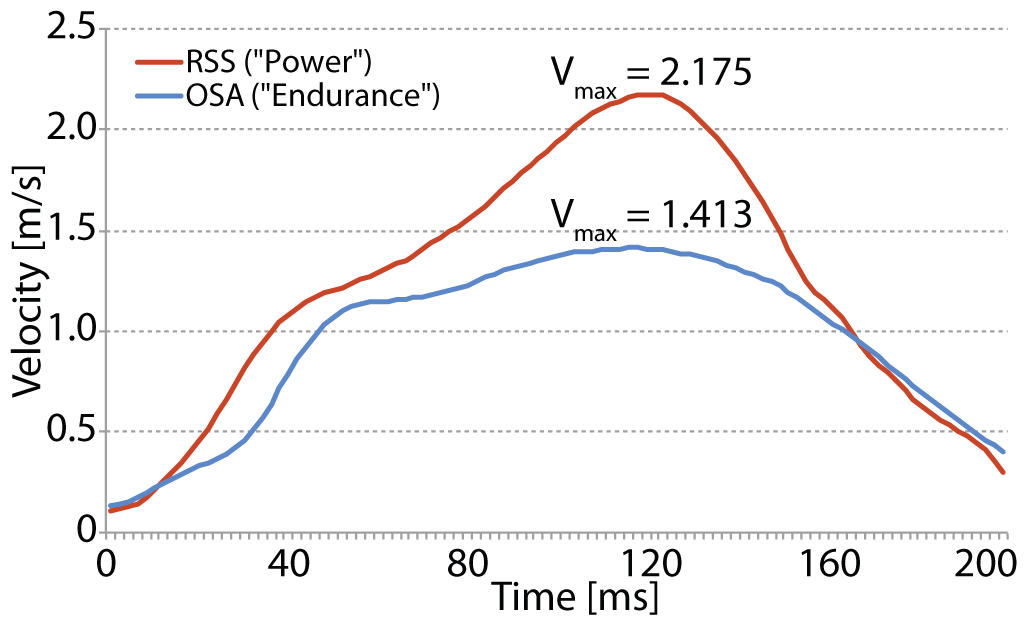
Exemplary kinematic patterns of plantarflexion. Note that subject RSS reaches a higher PF-v_max_ though range of motion (RSS: 62.5°; OSA: 59.2°) and peak acceleration (RSS: 36.68 m/s^2^; OSA: 35.40 m/s^2^) is pretty similar between both subjects.

**Figure 2:**
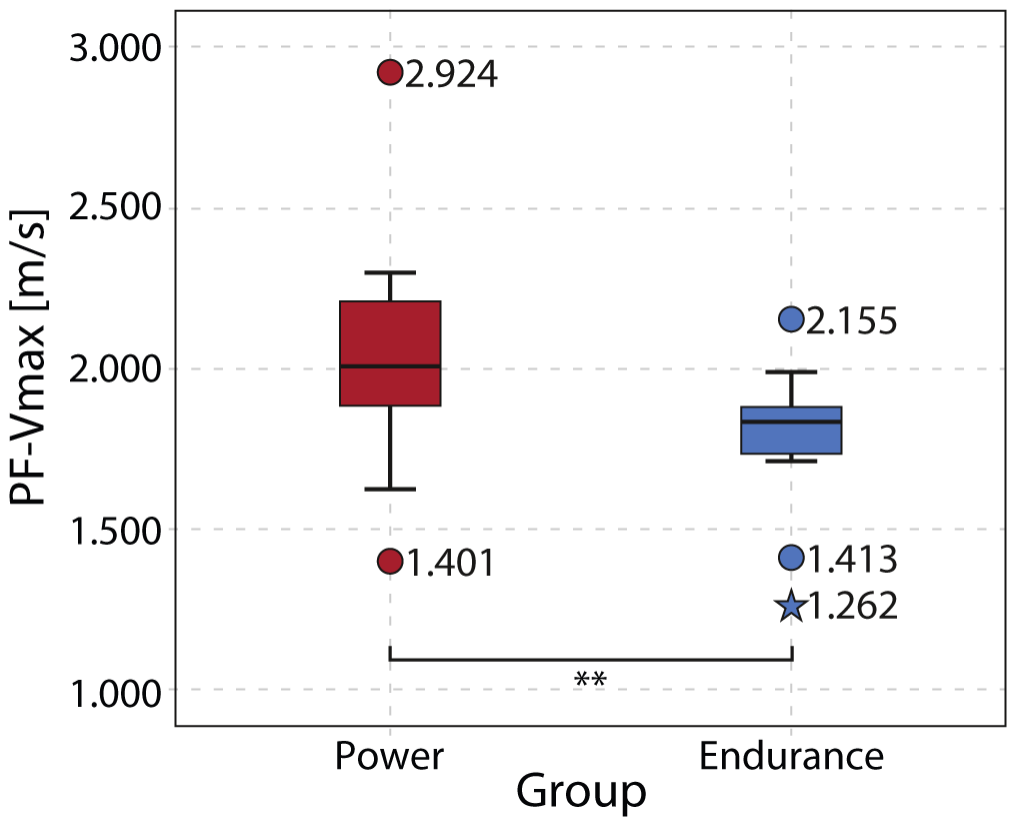
Maximum speed of an unloaded plantarflexion in power and endurance athletes. There is a significant difference between power and endurance athletes.

In line with our prediction, interindividual differences observed in PF-v_max_ were found in similar ranges of motion during foot movements (Figure 1). Accordingly, a significantly higher PF-v_max_ (p = 0.007) was found for the power athletes (2.03±0.34 m/s) compared to the endurance athletes (1.79±0.20 m/s). The effect size *r* = 0.38 can be considered as moderate (Figure 2).

**Figure 3:**
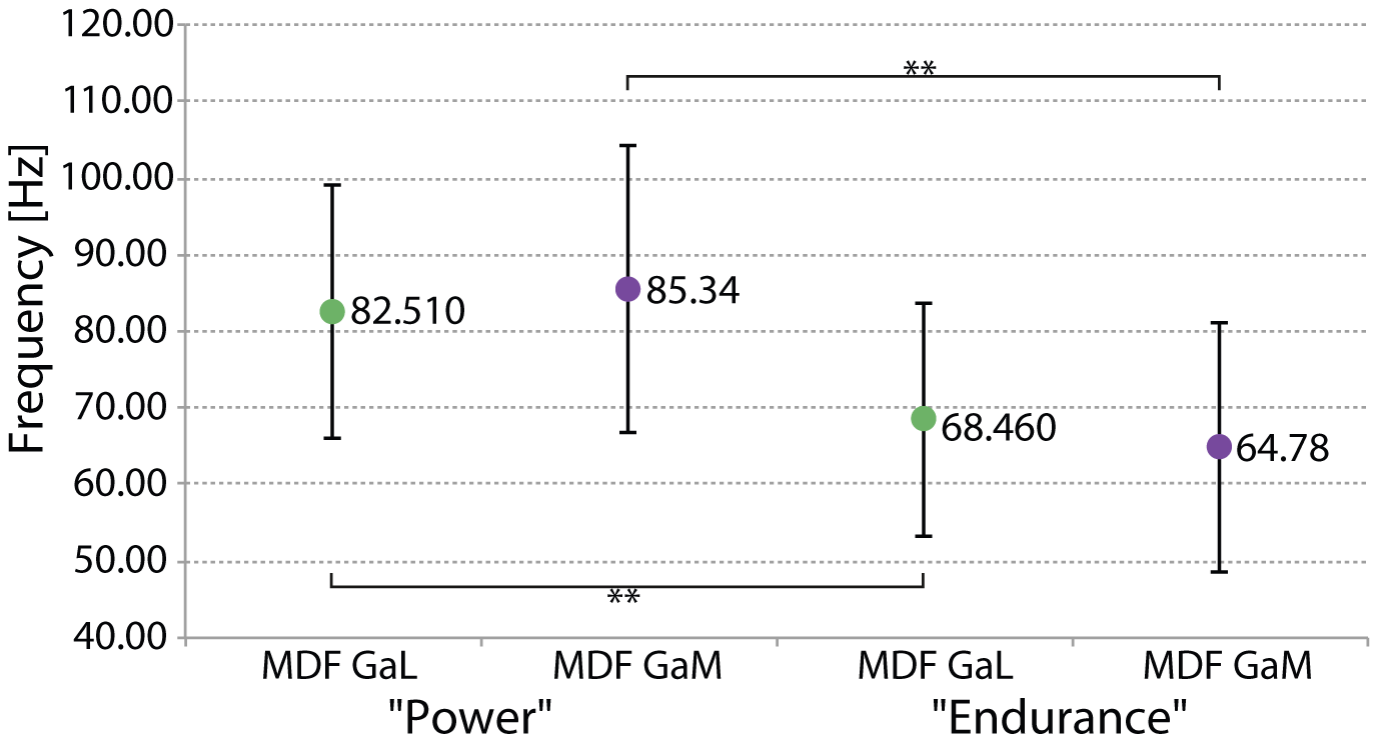
Group specific presentation of EMG Median frequencies (MDF) for power and endurance athletes. The mean MDF and standard deviation is depicted separately for the lateral (GaL) and medial (GaM) head of the gastrocnemius muscle. There are significant differences between power and endurance athletes.

Concerning EMG-data, we found a significant difference between both groups. The Median frequency (MDF) of the gastrocnemius lateralis muscle (MGL) as well as the gastrocnemius medialis muscle (MGM) were significantly higher for the power athletes (MDF GAL = 82.51±16.61 Hz; MDF GAM = 85.34±18.73 Hz) compared to their endurance counterparts (MDF GAL = 68.46±15.22 Hz; p = 0.009; MDF GAM = 64.78±16.19 Hz; p = 0.001). The effect size (GAL: *r* = 0.38; GAM: *r* = 0.46) can be considered as moderate (Figure 3). There was no relation between MDF and PF-v_max_ (Spearman's r = 0,239 (GaL); r = 0.208 (GaM), p>0.05).

### Imaging results

#### Functional localizer

**Figure 4:**
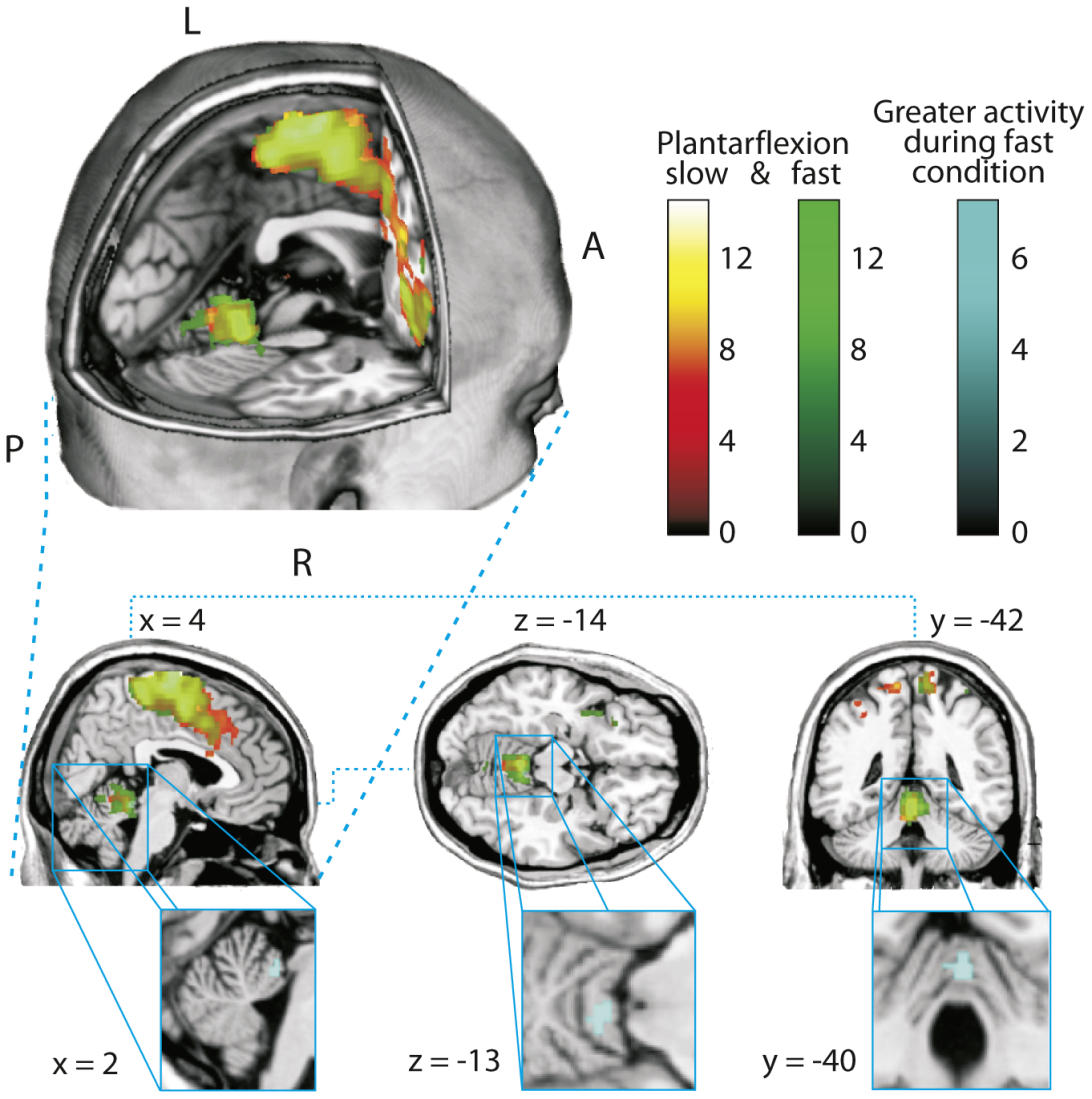
Brain activity during repeated dorsi- and plantarflexions with the left foot. The red-yellow spectrum indicates the slow condition and the fast condition is marked green (One sample t-test with FWE corrected threshold and p≤0.05). The significant greater activity during the fast condition is indicated light blue (Paired t-test with FWE corrected threshold and p≤0.05) and the corresponding brain region depicted separately increased. Note that the green colour (fast condition) in the motor cortex is almost completely overlaid with the red-yellow spectrum (slow condition).

Functional MRI analysis revealed contralateral brain activity in the supplementary (SMA) and primary motor cortex (M1) during repeatedly dorsi- and plantarflexions. For the slow condition local maxima with the coordinates x = 6.0; y = −12.0; z = 78.0 and x = 10.0; y = −36.0; z = 70.0 (FWE corrected) agree well with the expected lower leg and foot representation of the sensory-motor homunculus [32]. Similar results were obtained for the fast condition with local maxima at x = 4.0; y = −12.0; z = 74.0 (ROI_SMA_) and x = 6.0; y = −36.0; z = 70.0 (ROI_M1_). In both conditions, the anterior lobe of the cerebellum was significantly activated (slow: x = 0.0; y = −44.0; z = −14.0; fast: x = −2.0; y = −44.0; z = −12.0; FWE corrected). This cerebellar activity seems to be increased for the fast condition. Indeed, the paired t-test revealed a significantly (FWE corrected, p≤0.05) increased activity in the cerebellar vermis of the anterior lobe (ROI_Cereb_) with a local maximum at x = 4.0; y = −42.0; z = −14.0 (Figure 4; Table 1). Surprisingly, no such difference could be found for the supplementary or primary motor cortex. 2×2 ANOVA with factors group (“endurance” and “power”) and condition (“slow” and “fast”) indicated no effects of interaction, which means that the increased cerebellar activity for the fast condition is independent of the group.

**Table 1:** Areas of significantly greater activity.

Condition	Anatomical location	Voxel	Center (x, y, z)	t-statistic	Z-value
slow	Right SMA	17979	6, −12, 78	15.98	>8
	Left Cerebellum	838	−8, −36, −24	13.15	7.30
	Left Supra Marginal Gyrus	1949	−44, −36, 28	10.24	6.49
fast	Right SMA	8554	4, −12, 74	16.61	>8
	Left Cerebellum (Lobules I–IV)	1789	−12, −36, −24	16.53	>8
	Left Insula Lobe	4562	−42, 4, −2	14.90	7.68
Difference fast > slow	Cerebellar Vermis (Lobules I–IV)	58	4, −42, −14	7.28	5.52

Activity during slow and fast foot movements versus resting activity level and during fast condition compared to the slow condition. Clusters shown passed a FWE-corrected threshold with p≤0.05.

#### Structural analysis

**Figure 5:**
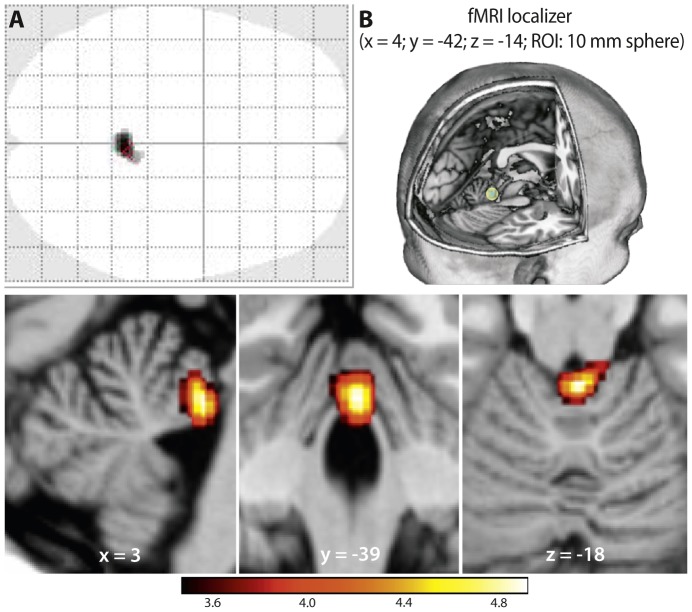
A: Whole brain analysis of structural differences between power and endurance athletes. (uncorrected voxel-level threshold; p≤0.001). B: ROI analysis; The power athletes show a significant higher volume of grey matter in the ROI_Cereb_ (Two Sample t-Test; p = 0.002; FWE corrected). The peak voxel (x = 3; y = −39; z = −18) is 7.65 mm remote from the functional peak voxel.

Structural MRI analysis was conducted by using a ROI analysis of speed-specific regions in the anterior cerebellum. According to the fMRI localizer results, we tested for significance in a 10 mm radius sphere around the peak coordinates x = 4.0; y = −42.0; z = −14.0 (ROI_Cereb_). This analysis revealed a significant higher volume of grey matter for the power athletes compared to the endurance athletes (FWE corrected). The peak voxel is located at x = 3.0; y = −39.0; z = −18.0 and is therefore positioned 7.65 mm away from the functional peak voxel (Figure 5 B). No such difference could be found in the ROI_M1_ or ROI_SMA_. Besides the functional and structural relationship, more evidence indicates a speed relevance of the cerebellar anterior lobe. Due to the functional localizer and to address the problem of multiple testing, we tested the relationship between maximum velocity of plantarflexion and grey matter volume in a procedure with hierarchical ranking [33]. We found a significant correlation between grey matter volume within the denoted sphere and the maximum velocity in the plantarflexion test (r = 0.30; p = 0.045; one tailed). No such correlation could be found for the grey matter volume in ROI_M1_ (r = 0.18; p = 0.33; two tailed) or ROI_SMA_ (r = 0.12; p = 0,51; two tailed) and PF-V_max_.

To show statistical tendencies for comparisons between power and endurance athletes, we looked for regions across the whole brain which might differ between both groups. Using an uncorrected voxel-level threshold with p≤0.001 the only difference in grey matter volume remains the described region in the cerebellar anterior lobe. No other significant differences could be detected (Figure 5 A).

## Discussion

The main purpose of this study was to identify differences in grey matter volume of the human brain concerning motor speed production in skilled sport athletes. As a model for motor speed production, we show increased activation of the cerebellar anterior lobe for ballistic plantarflexions (PF-Test) of the left foot. This speed-specific brain region shows higher volumes of grey matter in power-trained athletes as compared to endurance athletes. A weak correlation between the ROI's grey matter volume and maximum movement velocity of plantarflexion indicates that cerebellar grey matter volume is able to explain parts of variances in motor speed production. Our findings underline the importance of the cerebellum in regulating motor speed performance which plays a major role in competitive sports as well as in daily life to prevent accidents and injuries.

Motor speed is mainly determined by the central nervous and neuromuscular system [34], [35]. The demands for speed are sport-specific and differ above all between the so called power and endurance disciplines. For this reason we tested maximum movement velocity of plantarflexion in endurance and power trained athletes. As we expected, interindividual differences in maximum speed of plantarflexion could be seen and power athletes performed significantly better compared to their endurance counterparts. These findings are in accordance with other studies which tested maximum speed of single joint movements with no or just minor additional load [36]. The ability to produce high movement velocities is thought to be influenced by several factors like biomechanical and morphological features of the locomotor system [35], [37]. Besides that, the peripheral and central nervous system is a crucial influencing factor in terms of how the muscular system becomes innervated. Although a tetanic contraction is already reached when the motor unit's fire with approximately 60 Hz, much higher frequencies occur and lead to a faster increase of strength [38]. The relationship between motor units firing frequency and movement velocity has been proved in cats [39] as well as in humans [40]. Moreover, it is known that power training induces an increase of motor unit's firing frequency, e.g. in the dimension of 60 to 130 Hz [16]. Although we did not measure firing frequency directly, the frequency content of the EMG-signal allows us to estimate the muscle fibre recruitment pattern and the selection of muscle fibre types [28], [41]. Indeed, studies were able to demonstrate the dependence of median frequency in the EMG-signal and muscle fibre proportion, whereas FT-fibre percentage correlated positively with frequency parameters [42], [43]. More evidence is provided from studies which report simultaneously increases of movement velocity and EMG frequency components [44]–[46]. Our results from EMG analysis confirm these findings and show significantly higher median frequencies of gastrocnemius muscle during plantarflexion for the power athletes compared to the endurance athletes. We interpret this result as the ability of the power athletes to generate higher discharge rates of the motor units. Moreover, it supports the assumption that power athletes exhibit a higher portion of FT-fibres whereas endurance athletes are endued with more ST-fibres [47], [48]. On the one hand, high firing frequencies ensure the activation of high threshold fibres (FT-fibres) and on the other hand it helps to maximize the voluntary muscle activation [2], [49]. Particularly, the FT-fibre portion and the adequate innervation play a dominant role in ballistic contractions and it is likely that these aspects are responsible for the higher movement velocities measured in the power group. However, we could not find a correlation between MDF and PF-V_max_. This aspect supports Farina and colleagues (2002) who assume that frequency components are more a sign of recruiting new motor units than an indicator of central nervous control [50]. Nevertheless animal studies have shown correlations between discharge rate of motor cortex neurons or Purkinje cells and movement velocity of the forelimb [6], [7], [11], [12]. It is likely that such a relationship also exists for the foot and ankle. Due to the fact that neuronal discharge rate is in part dependent on the spatial summation at the dendritic tree, we hypothesized that speed specific brain regions show enlarged grey matter volume. In investigating this assumption, the first challenge we encountered was the identification of speed specific brain regions. According to former studies which characterized brain activity during repeated dorsi- and plantarflexions, we expected activity in the lower leg and foot representation of the supplementary and primary motor cortex of the contralateral brain hemisphere [32], [51], [52]. Besides that a bilateral subcortical activity could have been expected in the putamen, thalamus and cerebellum [51], [52]. Independent of condition we most notably found brain activity in the supplementary motor cortex, the primary motor cortex and in the cerebellum, which is in accordance with the studies mentioned above. Our results support the assumption that especially the cerebellum is involved in motor control and that certain populations of Purkinje cells encode movement velocity [9], [12], [53]. Functional activity in the cerebellum during repeated foot movements agrees with the cerebellar homunculus within the anterior lobe [54]. Along with the study of Hewitt and colleagues (2011) our results suggest that the ability to move as fast as possible is bound to the capability of generating highest possible discharge rates in a certain population of Purkinje cells [12]. Due to the concept of spatial summation at the dendritic tree, which in part underlies volumetric differences [55], it is likely that this ability is manifested in a neuroanatomical correlate of increased grey matter volume in the cerebellar anterior lobe. To the best of our knowledge this is the first time that a neurostructural correlate of motor speed is presented as one of the physical abilities.

Neuroimaging studies of professional musicians or athletes have demonstrated differences between experts and novices, which may reflect distinct training demands [19], [21], [22], [56], [57]. Although limited to our cross sectional design, we interpret our findings as structural and functional adaptations in consequence of long-term training. Motor and sporting activity induces structural changes in the central nervous as well as the neuromuscular system, which in return can influence the behaviour or performance with lasting effect. Thus it could be shown that training and improvement in a motor task leads to structural changes of task-specific brain regions [23], [58]. Besides tasks which emphasize the aspect of motor learning, training of physical abilities like strength also leads to neuronal adaptations, which can be detected e.g. by transcranial magnetic stimulation [59], [60]. Taking into account that the firing frequency of motor units influences the muscle fibre proportion [18], [61] differences between power and endurance athletes become more accountable. We hypothesize that power training with the permanent demand of explosive contractions leads to the ability to generate higher firing frequencies due to the enlarged grey matter volume in the cerebellar anterior lobe. This adaptation might be in part responsible for the differences in muscle fibre proportion which is modifiable and not solely genetically determined [62]. However, the muscle fibre alteration is thought to be a long-term process [47] and the supposed differences between the investigation groups might be a result of many years of training. In contrast to most longitudinal training studies, which found changes in cortical brain areas after short periods of practice [1], the present result identified a cerebellar brain region. We interpret the data observed in the power athletes as a training-induced neurostructural feature, which comes along with the ability to generate high discharge rates and which permits muscle contractions as fast as possible. To experimentally test this hypothesis, the training induced reorganization of cortico-cerebellar networks, the convertibility of motoneuronal firing frequency, the plasticity of muscle fibre proportion and the trainability of voluntarily initiated speed needs to be addressed in future longitudinal studies with training periods over several years. Furthermore it would be worthwhile to conduct comparable studies with a greater sample size. In the presented study, the small number of subjects in each group is a limiting factor, which was owed by the small pool of performance athletes available.

## Conclusions

While the exact mechanisms that underlie motor speed production in the peripheral and central nervous system remain unclear, our results show speed-specific neuro-functional and -structural correlates. Fast plantarflexions are accompanied by increased activity in the cerebellar anterior lobe and the same region shows increased grey matter volume for power compared to endurance athletes. Our findings are consistent with prior studies concerning motor speed and add evidence of a unique structural feature in the brains of speed-trained athletes.

## References

[1] Dayan E, Cohen LG (2011) Neuroplasticity Subserving Motor Skill Learning. Neuron 72: 443–454. 22078504 10.1016/j.neuron.2011.10.008 PMC3217208

[2] Hennemann E, Somjen G, Carpenter DO (1965) Functional Significance of Cell Size in Spinal Motoneurons. J Neurophysiol 28: 560–580. 14328454 10.1152/jn.1965.28.3.560

[3] Sale DG (1987) Influence of Exercise and Training on Motor Unit Activation. Exercise and Sport Sciences Reviews 15: 95–151. 3297731

[4] Mero A (1985) Relationships between the muscle fiber characteristics, sprinting and jumping of sprinters. Biology of Sport 2: 155–162.

[5] Andersen LL, Andersen JL, Magnusson SP, Suetta C, Madsen JL, et al (2005) Changes in the human muscle force-velocity relationship in response to resistance training and subsequent detraining. Journal of Applied Physiology 99: 87–94. 15731398 10.1152/japplphysiol.00091.2005

[6] Hamada I (1981) Correlation of monkey pyramidal tract neuron activity to movement velocity in rapid wrist flexion movement. Brain Research 230: 384–389. 6797678 10.1016/0006-8993(81)90420-0

[7] Schwartz AB (1993) Motor cortical activity during drawing movements: population representation during sinusoid tracing. Journal of Neurophysiology 70: 28–36. 8360717 10.1152/jn.1993.70.1.28

[8] Turner RS, Desmurget M, Grethe J, Crutcher MD, Grafton ST (2003) Motor Subcircuits Mediating the Control of Movement Extent and Speed. J Neurophysiol 90: 3958–3966. 12954606 10.1152/jn.00323.2003

[9] Turner RS, Grafton ST, Votaw JR, Delong MR, Hoffman JM (1998) Motor Subcircuits Mediating the Control of Movement Velocity: A PET Study. J Neurophysiol 80: 2162–2176. 9772269 10.1152/jn.1998.80.4.2162

[10] Ebner TJ, Hewitt AL, Popa LS (2011) What Features of Limb Movements are Encoded in the Discharge of Cerebellar Neurons? Cerebellum 10: 683–693. 21203875 10.1007/s12311-010-0243-0 PMC3711690

[11] Mano NI, Yamamoto KI (1980) Simple-spike activity of cerebellar Purkinje Cells related to visually guided wrist tracking movement in the monkey. Journal of Neurophysiology 43: 713–728. 6768848 10.1152/jn.1980.43.3.713

[12] Hewitt AL, Popa LS, Pasalar S, Hendrix CM, Ebner TJ (2011) Representation of limb kinematics in purkinje cell simple spike discharge is conserved across multiple tasks. J Neurophysiol 106: 2232–2247. 21795616 10.1152/jn.00886.2010 PMC3214117

[13] McNaughton S, Timmann D, Watts S, Hore J (2004) Overarm throwing speed in cerebellar subjects: effect of timing of ball release. Exp Brain Res 154: 470–478. 14579002 10.1007/s00221-003-1677-0

[14] Boreham C (2006) The physiology of sprint and power training. In: Whyte G, editors. The physiology of Training. Edinburgh, London, New York, Oxford, Philadelphia, St. Louis, Sydney, Toronto: Churchill Livingstone Elsevier. pp. 117–134.

[15] Shave R, Franco A (2006) The physiology of endurance training. In: Whyte G, editors. The physiology of Training. Edinburgh, London, New York, Oxford, Philadelphia, St. Louis, Sydney, Toronto: Churchill Livingstone Elsevier. pp. 61–84.

[16] Van Cutsem M, Duchateau J, Hainaut K (1998) Changes in single motor unit behaviour contribute to the increase in contraction speed after dynamic training in humans. Journal of Physiology 513: 295–305. 9782179 10.1111/j.1469-7793.1998.295by.x PMC2231276

[17] Gorza L, Gundersen K, Lomo T, Schiaffino S, Westgaard RH (1988) Slow-to-fast transformation of denervated soleus muscles by chronic high-frequency stimulation in the rat. Journal of Physiology 402: 627–649. 3236251 10.1113/jphysiol.1988.sp017226 PMC1191913

[18] Pette D, Vrbová G (1999) What does chronic electrical stimulation teach us about muscle plasticity? Muscle & Nerve 22: 666–677. 10366220 10.1002/(sici)1097-4598(199906)22:6<666::aid-mus3>3.0.co;2-z

[19] Gaser C, Schlaug G (2003) Brain Structures Differ between Musicians and Non-Musicians. The Journal of Neuroscience 23: 9240–9245. 14534258 10.1523/JNEUROSCI.23-27-09240.2003 PMC6740845

[20] Park IS, Lee KJ, Han JW, Lee NJ, Lee WT, et al (2009) Experience-Dependent Plasticity of Cerebellar Vermis in Basketball Players. Cerebellum 8: 334–339. 19259755 10.1007/s12311-009-0100-1

[21] Di X, Zhu S, Jin H, Wang P, Ye Z, et al (2012) Altered Resting Brain Function and Structure in Professional Badminton Players. Brain Connectivity 2: 225–233. 22840241 10.1089/brain.2011.0050 PMC3621728

[22] Wang B, Yuanyuan F, Lu M, Li S, Song Z, et al (2013) Brain anatomical networks in world class gymnasts: A DTI tractography study. NeuroImage 65: 476–487. 23073234 10.1016/j.neuroimage.2012.10.007

[23] Draganski B, Gaser C, Busch V, Schuierer G, Bogdahn U, et al (2004) Changes in grey matter induced by training. Nature 427: 311–312. 14737157 10.1038/427311a

[24] Taubert M, Lohmann G, Margulies DS, Villringer A (2011) Long-term effects of motor training on resting-state networks and underlying brain structure. NeuroImage 57: 1492–1498. 21672633 10.1016/j.neuroimage.2011.05.078

[25] Latash ML (2008) Neurophysiological Basis of Movement. Champaign, IL: Human Kinetics. 270 p.

[26] Harrison AJ, Keane SP, Coglan J (2004) Force-velocity relationship and stretch-shortening cycle function in sprint and endurance athletes. Journal of Strength and Conditioning Research 18: 473–479. 15320647 10.1519/13163.1

[27] Lattier G, Millet GY, Maffiuletti NA, Babault N, Lepers R (2003) Neuromuscular Differences Between Endurance-Trained, Power-Trained, and Sedentary Subjects. Journal of Strength and Conditioning Research 17: 514–521. 12930179 10.1519/1533-4287(2003)017<0514:ndbepa>2.0.co;2

[28] Basmajian JV, De Luca CJ (1985) Muscles Alive. Their Functions Revealed by Electromyography. Baltimore: Williams & Wilkins. 389 p.

[29] Sawilowsky SS (2005) Misconceptions Leading to Choosing the t Test Over the Wilcoxon Mann-Whitney Test for Shift in Location Parameter. Journal of Modern Applied Statistical Methods 4: 598–600.

[30] Power JD, Barnes KA, Snyder AZ, Schlaggar BL, Petersen SE (2012) Spurious but systematic correlations in functional connectivity MRI networks arise from subject motion. NeuroImage 59: 2142–2154. 22019881 10.1016/j.neuroimage.2011.10.018 PMC3254728

[31] Ashburner J (2007) A fast diffeomorphic image registration algorithm. NeuroImage 38: 95–113. 17761438 10.1016/j.neuroimage.2007.07.007

[32] Lotze M, Erb M, Flor H, Huelsmann E, Godde B, et al (2000) fMRI evaluation of somatotopic representation in human primary motor cortex. NeuroImage 11: 473–481. 10806033 10.1006/nimg.2000.0556

[33] Victor A, Elsäβer A, Hommel G, Blettner M (2010) Judging a Plethora of p-Values. Deutsches Ärzteblatt International 107: 50–56. 20165700 10.3238/arztebl.2009.0050 PMC2822959

[34] Seagrave L (2000) Neuro-Biomechanics of Maximum Velocity. IAAF regional development centre bulletin 17: 21–26.

[35] Cronin JB, McNair PJ, Marshall RN (2003) Force-velocity Analysis of Strength-Training Techniques and Load: Implications for Training Strategy and Research. Journal of Strength and Conditioning Research 17: 148–155. 12580670 10.1519/1533-4287(2003)017<0148:fvaost>2.0.co;2

[36] Wrbaškiæ N, Dowling JJ (2009) The relationship between strength, power and ballistic performance. Journal of Electromyography and Kinesiology 92: e14–e22. 10.1016/j.jelekin.2007.07.011 18035556

[37] Sabrina SM, Piazza SJ (2009) Built for speed: musculoskeletal structure and sprinting ability. The Journal of Experimental Biology 212: 3700–3707. 19880732 10.1242/jeb.031096

[38] Sale DG (2003) Neural Adaptations to Strength Training. In: Komi PV, editors. Strength and Power in Sport. Oxford: Blackwell Science Ltd. pp. 281–314.

[39] Hoffer JA, O'Donovan MJ, Pratt CA, Loeb GE (1981) Discharge patterns of hindlimb motoneurons during normal cat locomotion. Science 213: 466–468. 7244644 10.1126/science.7244644

[40] Grimby L (1984) Firing properties of single human motor units during locomotion. J Physiol 346: 195–202. 6699774 10.1113/jphysiol.1984.sp015016 PMC1199493

[41] van Tscharner V, Goepfert B (2003) Gender dependent EMGs of runners resolved by time/frequency and principal pattern analysis. Journal of Electromyography and Kinesiology 13: 253–272. 12706605 10.1016/s1050-6411(02)00111-6

[42] Gerdle B, Henriksson-Larsen K, Lorentzon R, Wretling ML (1991) Dependence of the mean power frequency of the electromygram on muscle force and fibre type. Acta Physiol Scand 142: 457–465. 1835248 10.1111/j.1748-1716.1991.tb09180.x

[43] Kupa EJ, Roy SH, Kandarian SC, De Luca CJ (1995) Effects of muscle fiber type and size on EMG median frequency and conduction velocity. Journal of Applied Physiology 79: 23–32. 7559225 10.1152/jappl.1995.79.1.23

[44] Tamaki H, Kitada K, Akamine T, Sakou T, Kurata H (1997) Electromyogram patterns during plantarflexions at various angular velocities and knee angles in human triceps surae muscles. European Journal of Applied Physiology 75: 1–6. 10.1007/s004210050118 9007450

[45] Hutchins R, Miller JP, Croce R (1998) Effect of movement velocity on the median frequency of the electromyographic activity of the quadriceps and hamstrings during isokinetic testing. Isokinetics and Exercise Science 7: 75–78.

[46] Hodson-Tole EF, Wakeling JM (2008) Motor unit recruitment patterns 1: responses to changes in locomotor velocity and incline. The Journal of Experimental Biology 211: 1882–1892. 18515718 10.1242/jeb.014407

[47] Andersen JL, Schjerling P, Saltin B (2000) Muscle, Genes and Athletic Performance. Scientific American 48–55. 10.1038/scientificamerican0900-48 10976466

[48] Aagaard P, Bangsbo J (2006) The Muscular System: Design, Function, and Performance Relationships. In: Tipton CM, Sawka MN, Tate CA, Terjung RL, editors. ACSM's Advanced Exercise Physiology. Philadelphia, Baltimore, New York, London, Buenos Aires, Hong Kong, Sydney, Tokyo: Lippincott Williams & Wilkins. pp. 144–160.

[49] Kernell D (1993) The gradation of muscle force and the functional organisation of spinal motoneurons and motor units. In: Bakker CMC, Berger MAM, Doorenbosch CAM, Peper CE, Willems MET, et al.., editors. Neural Aspects of Human Movement. Implications for control and coordination. Amsterdam, Lisse, Berwyn: Swets & Zeitlinger. pp. 12–22.

[50] Farina D, Fosci M, Merletti R (2002) Motor unit recruitment strategies investigated by surface EMG variables. Journal of Applied Physiology 92: 235–247. 11744666 10.1152/jappl.2002.92.1.235

[51] Newton JM, Dong Y, Hidler J, Plummer-D'Amato P, Marehbian J, et al (2008) Reliable assessment of lower limb motor representations with fMRI: Use of a novel MR compatible device for real-time monitoring of ankle, knee and hip torques. NeuroImage 43: 136–146. 18675363 10.1016/j.neuroimage.2008.07.001 PMC2658811

[52] Trinastic JP, Kautz SA, McGregor K, Gregory C, Bowden M, et al (2010) An fMRI study of the differences in brain activity during active ankle dorsiflexion and plantarflexion. Brain Imaging and Behavior 4: 121–131. 20502995 10.1007/s11682-010-9091-2

[53] Coltz JD, Johnson MTV, Ebner TJ (1999) Cerebellar Purkinje Cell Simple Spike Discharge Encodes Movement Velocity in Primates during Visuoumotor Arm Tracking. The Journal of Neuroscience 19: 1782–1803. 10024363 10.1523/JNEUROSCI.19-05-01782.1999 PMC6782164

[54] Schlerf JE, Verstynen TD, Ivry RB, Spencer RMC (2010) Evidence of a Somatotopic Map in the Human Neocerebellum During Complex Actions. J Neurophysiol 103: 3330–3336. 20393055 10.1152/jn.01117.2009 PMC2888250

[55] Anderson BJ (2011) Plasticity of Gray Matter Volume: The Cellular and Synaptic Plasticity That Underlies Volumetric Change. Developmental Psychobiology 53: 456–465. 21678393 10.1002/dev.20563

[56] Yarrow K, Brown P, Krakauer W (2009) Inside the brain of an elite athlete: the neural processes that support high achievement in sports. Nature Reviews Neuroscience 10: 585–596. 19571792 10.1038/nrn2672

[57] Jäncke L, Langer N, Hänggi J (2012) Diminished whole-brain but enhanced peri-sylvian connectivity in absolute pitch musicians. Journal of Cognitive Neuroscience 24: 1447–1461. 22524277 10.1162/jocn_a_00227

[58] Taubert M, Draganski B, Anwander A, Müller K, Horstmann A, et al (2010) Dynamic properties of human brain structure: Learning-related changes in cortical areas and associated fiber connections. The Journal of Neuroscience 30: 11670–11677. 20810887 10.1523/JNEUROSCI.2567-10.2010 PMC6633410

[59] Carroll TJ, Riek S, Carson RG (2002) The sites of neural adaptation induced by resistance training in humans. J Physiol 544: 641–652. 12381833 10.1113/jphysiol.2002.024463 PMC2290590

[60] del Olmo MF, Reimunde P, Viana O, Acero RM, Cudeiro J (2006) Chronic neural adaptation induced by long-term resistance training in humans. European Journal of Applied Physiology 96: 722–728. 16506058 10.1007/s00421-006-0153-5

[61] Buller AJ, Eccles JC, Eccles RM (1960) Interactions between motoneurons and muscles in respect of the characteristics speeds of their responses. J Physiol 150: 417–439. 13805874 10.1113/jphysiol.1960.sp006395 PMC1363172

[62] Simoneau JA, Bouchard C (1995) Genetic determinism of fiber type proportion in human skeletal muscle. The FASEB Journal 9: 1091–1095. 7649409 10.1096/fasebj.9.11.7649409

